# A Review of Point Set Registration: From Pairwise Registration to Groupwise Registration

**DOI:** 10.3390/s19051191

**Published:** 2019-03-08

**Authors:** Hao Zhu, Bin Guo, Ke Zou, Yongfu Li, Ka-Veng Yuen, Lyudmila Mihaylova, Henry Leung

**Affiliations:** 1 Key Laboratory of Industrial IoT and Networked Control, Ministry of Education, and the Key Laboratory of Intelligent Air-Ground Cooperative Control for Universities in Chongqing, College of Automation, Chongqing University of Posts and Telecommunications, Chongqing 400065, China; gbxxq666@163.com (B.G.); kezou18@163.com (K.Z.); liyongfu@cqupt.edu.cn (Y.L.); 2 Faculty of Science and Technology, University of Macau, Macau 999078, China; kvyuen@umac.mo; 3 Department of Automatic Control and Systems Engineering, University of Sheffield, Mappin Street, Sheffield S1 3JD, UK; L.S.Mihaylova@sheffield.ac.uk; 4 Department of Electrical and Computer Engineering, University of Calgary, 2500 University Drive NW, Calgary, AB T2N 1N4, Canada; leungh@ucalgary.ca

**Keywords:** point set registration, pairwise registration, groupwise registration

## Abstract

This paper presents a comprehensive literature review on point set registration. The state-of-the-art modeling methods and algorithms for point set registration are discussed and summarized. Special attention is paid to methods for pairwise registration and groupwise registration. Some of the most prominent representative methods are selected to conduct qualitative and quantitative experiments. From the experiments we have conducted on 2D and 3D data, CPD-GL pairwise registration algorithm and JRMPC groupwise registration algorithm seem to outperform their rivals both in accuracy and computational complexity. Furthermore, future research directions and avenues in the area are identified.

## 1. Introduction

Point set registration is a challenging aspect in pattern recognition [1,2,3,4,5], computer vision [6,7], robotics [8,9,10,11] and image processing [12,13,14]. For example, in medical image processing, in order to fuse multiple images by computed tomography (CT), magnetic resonance imaging (MRI) and positron emission tomography (PET), the fundamental step is to register the feature points from CT, MRI, and PET. In intelligent vehicles, pre-processing is an important step prior to feature points extraction from many sensors, such as radio detection and ranging (Radar), light detection and ranging (LiDAR) and camera. Point set registration methods [15,16] have then proposed to align the images and extract feature points that will be further used for localization and mapping. In face recognition, face landmarks are extracted from a face with different facial expressions or different viewpoints. Then, point set registration can be used to perform the task of face recognition [17].

**Figure 1:**
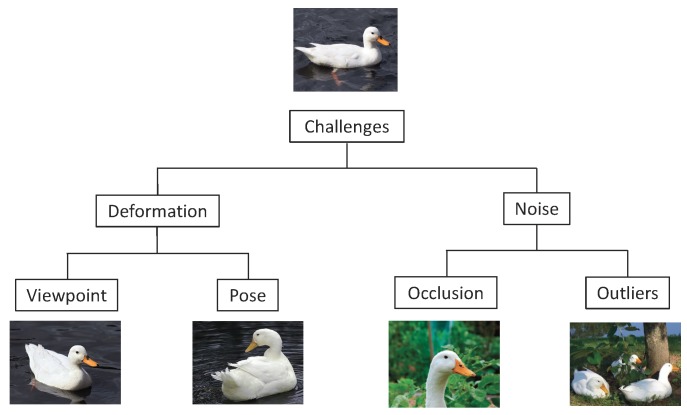
Some challenges in the point set registration [22].

The main purpose of the point set registration is to find correspondences and to estimate the transformation between two or more point sets. In practice, point set registration methods suffer from many challenges due to deformation and noise. Different viewpoints or different poses may cause the deformation between point sets. The noise between point sets includes occlusion and outliers. Missing points occur due to feature extraction in the case of occlusion. Outliers have no correspondence in the other point sets. These challenges are shown in Figure 1. Furthermore, high dimensionality and massive point sets are commonly encountered in the real world, e.g. about million points will be obtained by LiDAR scanning. The scale-invariant feature transform (SIFT) methods [18,19] have contributed to solving many challenging problems with LiDAR and other imagery data. Recently, some deep learning methods have also been developed to select the feature points from medical image and remote sensing image [20,21].

**Figure 2:**
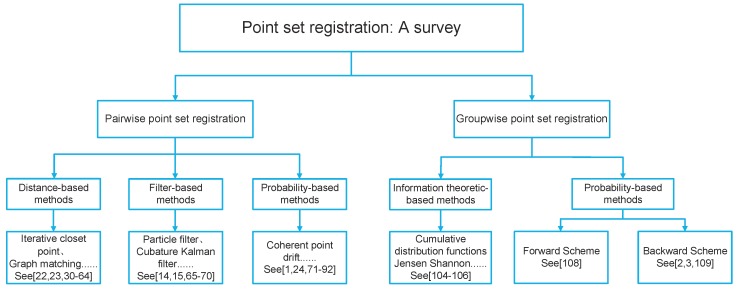
Taxonomy of point set registration methods.

Normally, the point set registration methods fall into two categories: pairwise and groupwise. Pairwise registration only considers two point sets while groupwise registration performs more than two point sets simultaneously. According to the modeling methods of point set registration, they can be categorized into parametric models and non-parametric models. Parametric models include the classic iterative closest point (ICP) method [23,24], and probabilistic point set registration using Gaussian mixture model (GMM) [25]. Graph matching (GM) is the traditional method in the non-parametric model [26]. According to the difference in transformation, the point set registration methods can be roughly classified into rigid transformation and nonrigid registration. The rigid transformation only considers translation, rotation, and scaling. The affine transformation, which is a nonrigid transformation, allows anisotropic scaling and skews [25]. Compared with rigid transformation, the nonrigid transformation is more challenging as the true nonrigid transformation model is often unknown [27,28]. The methods of point set registration are summarized in Figure 2.

The rest of the paper is organized as follows. Section 2 describes the pairwise point set registration methods. Section 3 reviews the groupwise point set registration methods. Some representative point set registration algorithms are selected to conduct experiments comparison in Section 4. Finally, Section 5 concludes the paper and gives the future trends and research avenues in this area.

## 2. Pairwise Point Set Registration

**Figure 3:**
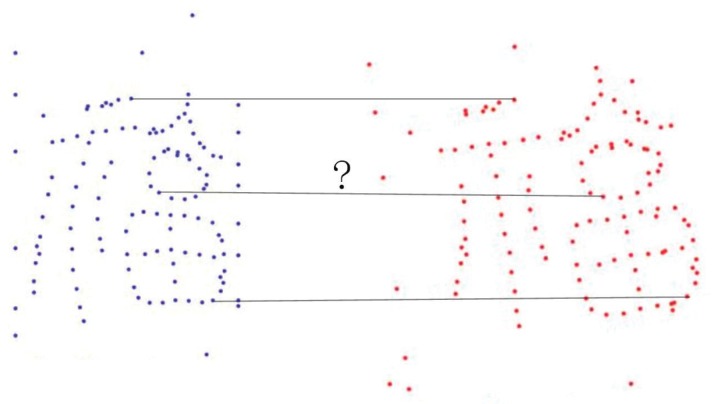
Pairwise point set registration problem: find the correspondences and the transformation of two point sets.

Considering two point sets $X={\left\{x_{i}\left|x_{i}\in {ℜ}^{D}\right.\right\}}_{i=1}^{N}$ and $Y={\left\{y_{j}\left|y_{j}\in {ℜ}^{D}\right.\right\}}_{j=1}^{M}$, where *D* denotes the dimension of these points. An example of pairwise point set registration is shown in Figure 3. The goal of pairwise point set registration methods is to find the suitable transformation and to establish the correct correspondences between X and Y. Many methods have been developed to address this problem. Some surveys on recent developments in pairwise point set registration can be found in [29,30]. These methods can be roughly classified into three categories: distance-based methods, filtering-based methods and probability-based methods.

### 2.1. Distance-Based Methods

The distance-based point set registration methods involve a dual-step scheme. The first step is to compute a distance between two point sets and to find the correspondences. Then, the distance between two point sets with the determined correspondences is minimized in the second step. The ICP, introduced by Besl and McKay [23] and Zhang [24], is the well-known method in the field of point set registration for rigid transformation between two points. The ICP can be expressed an optimization problem

(1) $$\underset{R,t}{\arg\min}\left\{\frac{1}{M}\sum_{j=1}^{M}{∥y_{j}-\left(Rx_{j}+t\right)∥}_{2}\right\}$$

where $x_{j}$ and $y_{j}$ is a correspondence pair, ${∥.∥}_{2}$ is the Euclidean norm, R and t are a rotation matrix and translation vector, respectively; and *M* is the number of correspondence pairs. Some surveys on recent developments in ICP method can be found in [31,32,33]. Many stages and efficient variants are given in the literature [31], such as selection of points, matching points, weighting of pairs, rejecting pairs, error metric and minimization, and high-speed variants. The aim of selection of point is to boost the convergence of ICP algorithm. The step of matching points is to find the correspondences between two point sets. Some methods have been proposed to assign the weights of correspondence [31], such as constant weight method, larger weight method, distance points method and smaller weight method. The purpose of rejecting pairs is to eliminate outliers for improving the performance of point set registration. Finally, the correspondence is computed using the current transformation and a new transformation is obtained by minimizing the sum of squared distances between the correspondence points.

However, the ICP method is sensitive to the initial conditions and can be trapped into local minima. A robust point matching (RPM) method [34] was proposed to solve this problem. RPM combines deterministic annealing and soft-assign optimization to convexify the objective function. However, the RPM method is restricted to perform rigid-body transformation. Therefore, a thin-plate spline robust point matching (TPS-RPM) method was developed in [35]. Deterministic annealing, soft-assign, thin-plate spline for spatial transformation and outlier rejection are used to perform both the correspondences and transformation parameters [35]. However, the TPS-RPM method can hardly be easily extended for higher dimension point sets.

In [36], a kernel correlation (KC) algorithm was proposed to align intensity images. KC is a function of point set entropy and an affinity measure. The point set registration is performed by maximizing the KC of point sets. In [37], point set registration was formulated by kernel density correlation metric, which is similar to the method in [36]. It is noted that a kernel function mainly determines the performance of point set registration in the KC method.

In [38], a GMMReg method was proposed to perform point set registration. Two point sets can be represented by two Gaussian mixture models (GMMs). The point set registration is considered as aligning the two GMMs. The Euclidean distance of two GMMs was minimized to estimate the transformation of two point sets. In [39], a support vector-parametrized Gaussian mixture (SVGM) method, which is an adaptive data representation method of point sets, was developed to improve the robustness to outliers, noises, and occlusions. In SVGM, the point set is represented by a one-class support vector model (SVM) and the output function is approximated by a GMM.

**Figure 4:**
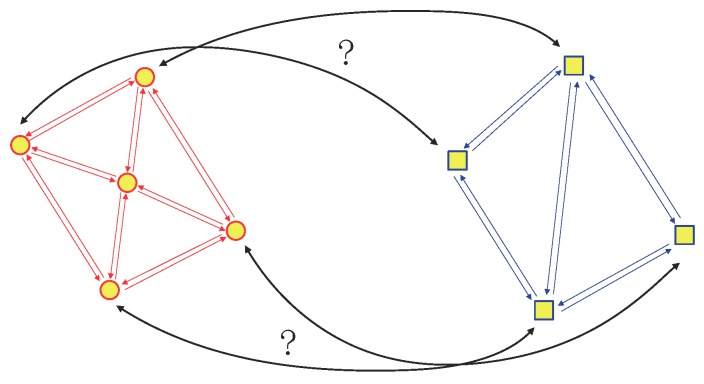
An example of GM problem.

Graph matching (GM) is a popular method in the point set registration using non-parametric model [26]. An example of GM is shown in Figure 4. A graph consists of some vertices and edges. GM methods find the correspondences between two graphs using the feature descriptors with vertexes and edges [40,41]. Some surveys in the GM method are given in [42,43]. GM can be considered as an optimization problem. The objective function of the optimization problem incorporates with vertices and edges of two graphs. In the form of objective function, the GM methods can be classified into three categories as first-order GM methods, second-order GM methods and high-order GM methods. First-order GM methods only consider the local feature descriptors with the information of vertexes. This idea is similar to ICP and its variants.

Most current GM algorithms are second-order or high-order GM methods [22,44,45,46,47,48,49,50,51,52,53,54,55,56,57,58,59,60,61,62,63,64,65]. Second-order GM methods combine the similarity of vertices-to-vertices and edges-to-edges. High-order GM methods involve the information of hyper-graph, which is hyper-edges incorporating the angles of tuples of vertices. The second-order or high-order GM methods are expressed as a quadratic assignment problem (QAP) [44].

Many second-order GM methods have been reported in the literature. In contrast to the linear assignment problem in first-order GM methods, which can be performed by the Hungarian algorithm, the QAP is an NP-hard problem [45]. Therefore, one issue of GM method is on the development of an accurate estimation algorithm. Many methods have been proposed to approximate the QAP problem. It can be classified into three categories: spectral relaxation, semi-definite programming relaxation, and doubly stochastic relaxation. In [46], a spectral relaxation was proposed to approximate the QAP problem, and then the spectral matching (SM) method was developed. In [47], a new SM method was incorporated with an affine constraint to provide a higher relaxation than the SM method. The semi-definite programming (SDP) relaxation is another method for approximating the QAP solution. The SDP methods relax the non-convex constraint using a convex semi-definite. The correspondence is approximated using a randomized algorithm [48] or a winner-take-all method [49]. Using a doubly stochastic matrix, the optimizing GM is transformed as a non-convex QAP problem. Therefore, many methods can be used to find a local optimum. In [50], the quadratic cost was approximated using a linear program, which was performed by a simplex-based algorithm. In [51], an integer projection algorithm was proposed to optimize the objective function in the integer domain. In [53], a probabilistic formulation of the SM method [46] was given. It estimates the assignment probabilities by maximum-likelihood. More recently, a factorized graph matching (FGM) method was developed in [22]. In FGM, the large pairwise affinity matrix was factorized into some smaller matrices. A path-following optimization algorithm was then proposed to improve the matching performance.

High-order GM methods involve high-dimensional of information of hyperedges. Third-order GM methods are usually considered. The advantage of high-order GM methods is that the high-order matching method is invariant to scale and affine changes. In [55], a probabilistic interpretation of high-order GM methods was formulated. In [56], the high-order matching problem was formulated as a tensor optimization problem. In [57], an high-order GM method was developed by adopting jumps with a reweighting scheme. In [58], a framework of tensor block coordinate ascent methods was proposed for high-order matching. Recently, In [65], a K-nearest-neighbor-pooling matching method, which integrates feature pooling into GM, was introduced for a second-order GM. A sub-pattern structure was then constructed for a high-order GM.

### 2.2. Filtering-Based Methods

The filter-based point set registration methods perform the point set registration using a state space model (SSM). In general, the SSM is formulated as:

(2) $$\begin{array}{c} {\tilde{x}}_{k}={\tilde{x}}_{k-1}+v_{k}\\ y_{k}=f\left({\tilde{x}}_{k},x_{k}\right)+w_{k} \end{array}$$

where $x_{k}$ and $y_{k}$ are the points from two sets; ${\tilde{x}}_{k}$ is the state at time *k*, and it can be written as ${\tilde{x}}_{k}=[t_{k}^{x},t_{k}^{y},\theta_{k}{]}_{}^{T}$ in 2D point sets; $t_{k}^{x}$ and $t_{k}^{y}$ are the translation parameters in x-axis and y-axis at time *k*, respectively; $\theta_{k}$ is the rotation parameter at time *k*. For 3D point sets, the state is denoted as ${\tilde{x}}_{k}=[t_{k}^{x},t_{k}^{y},t_{k}^{z},\theta_{k}^{x},\theta_{k}^{y},\theta_{k}^{z}{]}_{}^{T}$, where $t_{k}^{z}$ is the translation parameter in z-axis at time *k*, $\theta_{k}^{x}$, $\theta_{k}^{y}$, $\theta_{k}^{z}$ are the rotation parameters in the x-axis, y-axis and z-axis at time *k*, respectively; f(.) is the measurement function; $v_{k}$ and $w_{k}$ are the process noise and measurement noise, respectively; $v_{k}$ and $w_{k}$ are assumed to be zero-mean Gaussian white noise.

In [66], an unscented particle filter (UPF) was used for rigid registration. The ICP algorithm was used to find correspondences and to compute the distance between data sets. This method is not sensitive to outliers. In [67], a particle filter was proposed for point set registration. An iterative-based local optimizer, which can be reinterpreted as a robust version of ICP, was formulated based on the correlation measure. In [68], a deformable registration framework, composed of simulated annealing with a particle filter, was proposed to point set registration. A variety of constraints on the registration are incorporated into this method. Furthermore, a novel method to regularize the deformation field was proposed to improve the registration performance. In [69], a map was generated by fusing inertial measurement unit (IMU), odometry, global positioning system (GPS) and LiDAR. Live laser data were aligned with the prior-map using a particle filter based point set registration method. In [70], an unscented Kalman filter (UKF) method was proposed to register two data sets in the presence of noise. However, the correspondences of these two point sets were assumed known. In [15,16], a local shape descriptor was proposed to obtain the correspondences of point sets. A rigid point set registration method based on cubature Kalman filter (CKF) was presented for localization in the intelligent vehicle. In [71], the authors considered that noise, outliers, false initialization, and other errors might exist simultaneously. A split covariance intersection filter (SCIF) was then proposed to point set registration under a filtering framework.

### 2.3. Probability-Based Methods

The coherent point drift (CPD) [25] is a popular method in field of probability-based point set registration. In the CPD method, a rigid and non-rigid point set registration is formulated as a maximum likelihood (ML) estimation problem using GMM method. One point set is represented by GMM centroids, and the another point set is fitted to those of the first point set by moving coherently:

(3) $$p\left(Y\right)=\prod_{j=1}^{M}\sum_{i=1}^{N}\pi_{i}N\left(y_{j}\left|g\left(x_{i}\right),\sigma^{2}I_{D}\right.\right)$$

where N(.) is the Gaussian distribution; g(.) is the rigid or non-rigid transformation; $\sigma^{2}$ is the equal isotropic covariances; I is the identity matrix; and $\pi_{i}$ is the mixing coefficient. Then, an expectation-maximization (EM) algorithm is applied to perform this ML optimization. Many algorithms were proposed to extend the CPD method [1,72,73,74,75,76,77,78,79,80,81,82,83,84,85,86,87,88,89,90,91,92]. These algorithms can be summarized as follows:

(1) Selecting a suitable non-rigid transformation function: In the CPD method, only one non-rigid transformation function is considered. Therefore, multiple kernel functions were used to represent non-rigid transformations in [72]. By automatically adjusting the kernel weights, this method can prune the ineffective kernels and evaluate the importance of each kernel. Considering the multi-layer motion between two sets of points, a robust point set registration using the GMM model was proposed in [73].
(2) Choosing the distribution of point set: In [74], the Student’s-*t* distribution was used to replace the Gaussian distribution for tackling the outliers in the point set registration. Similar to the CPD method, one point set is treated as Student’s-t mixture model centroids, while another point set is fitted to those of the Student’s-t mixture model centroids by moving coherently.
(3) Setting the membership probabilities: In the CPD method, equal membership probabilities were used. To improve the performance of point set registration, the shape context was proposed to assign the membership probabilities of the mixture model in [1].
(4) Developing the local structure descriptors: In the CPD method, the GMM centroids were forced to move coherently to fit the data points by maximizing the likelihood, which only encodes the global structure of the two point sets. To preserve the local structure of point sets, the idea of local linear embedding (LLE) was proposed. The local neighbors in the point set could be preserved after the non-rigid transformed. Each point can be represented by a weighted linear of its neighbors. Then, an EM algorithm was derived for the ML optimization constrained with both CPD and LLE terms [75]. Similar to the LLE, the locally linear transforming (LLF) was developed for constructing the local structure [76]. In [1], the local features were used to assign the membership probabilities of the GMM. A non-rigid point set registration, which preserves both global and local structures, was developed. In [78], the shape context and LLF were proposed to the nonrigid point set registration. In [17], a non-rigid point set registration using spatially constrained Gaussian fields (SCGF) was developed. The shape context was also used for the membership probabilities initialization. A graph Laplacian regularized Gaussian fields was proposed to preserve the local structure of point sets. Furthermore, two local structure descriptors were embedded in the CPD framework in [79]. The first descriptor was LLE. The Laplacian coordinate was used in the second descriptor to keep the size of neighborhood structure. Therefore, the objective function of point set registration was composed of the global distance item, non-rigid transformation constraint item and two local structure constraints items.
(5) Extraction the feature of point sets: The spatial location of point sets is a traditional feature for registration. In [86], the color information of point sets was used to extend the CPD algorithm. In [87], the correlation of color information and spatial location information was formulated. Then, a probabilistic point set registration framework with color information and spatial location information was given.
(6) Performing algorithm: The disadvantage of the traditional CPD algorithm is that the CPD method has a high computation cost. Therefore, In [88], an accelerated CPD (ACPD) method was proposed to register a 3-D point cloud. In ACPD, a global squared iterative EM algorithm was developed to speed up the process of likelihood maximization. The dual-tree improved fast Gauss transform method was used to accelerate the process of Gaussian summation. In [92], the regression and clustering for performing point set registration in a Bayesian framework were presented. The coarse-to-fine variational inference algorithm was used to estimate the unknown parameters.

### 2.4. Discussion

As the distance-based methods possess acceptable performance and computation load, they are widely used in many fields, such as target tracking [93]. The filter-based methods have the capability to register the massive point set online. However, the correspondences of the point set should be computed in advance. From the literature, the probability-based methods with some local structures perform better than other methods, but the former have higher computation cost than the distance-based methods and the filter-based methods.

## 3. Groupwise Point Set Registration

Let $M_{j}=\left[M_{j1},M_{j2}...M_{jN_{j}}\right]$ be the *j*-th point set. Let $M={\left\{M_{j}\right\}}_{j=1}^{M}$ denote the union of multiple point sets, where *M* is the number of sets. One important issue is on the registration of these point sets. Traditionally, this problem is performed using pairwise registration repeatedly [2], such as sequentially strategy [94,95,96,97,98] and one-versus-all strategy [99,100,101]. In the sequentially pairwise registration strategy, the parameters are updated by a ICP method or a probabilistic method when additional point sets are available. The main drawback of sequentially pairwise registration strategy consists in the error propagation in the subsequent steps [2,3]. For the one-versus-all pairwise registration strategy, the reference point set should be chosen in advance. The other point sets are used to register with the reference point set.

Simultaneous registration of multiple point sets is another method which brings further improvement to the point set methods. They are called groupwise point set registrations. In [102], some correspondences between the point sets were assumed known in advance, and the transformation parameters were estimated. Furthermore, in [103], the same formulation as [102] was extended to perform unknown correspondences. The above literature also developed the simultaneous multiple point sets registration with a pairwise strategy. Some methods have been developed to register multiple point sets simultaneously without the resource of a pairwise strategy. It can be categorized as information theoretic-based methods and probability-based methods.

For information theoretic-based methods, the joint multiple point sets registration is performed according to some information theoretic measures. In [104], an information theoretic measure, which is named as cumulative distribution functions Jensen Shannon (CDF-JS) method, was proposed to register multiple point sets. As the CDF-JS method is symmetric and had no bias to any point sets, it can register the multiple point sets simultaneously. The cost function was defined as the CDF-JS divergence and was minimized by computing analytic gradients in a quasi-Newton scheme. However, this method has a high computation cost for the CDF-JS and has no closed-form solutions. In [105], another information theoretic measure, called cumulative distribution functions Havrda-Charvát (CDF-HC) method, was developed. The CDF-HC method uses the same idea as the CDF-JS method but with a different divergence for the cumulative distribution functions. In the CDF-HC method, the Havrda-Charvát divergence was proposed instead of Jensen Shannon divergence. Compared with CDF-JS method, the CDF-HC method is much simpler to implement and has lower computation cost [105]. Recently, a Rényi’s second order entropy method was proposed for groupwise point set registration in [106]. It is a closed-form solution to the cost function.

For probability-based methods, the multiple point sets are formulated as some probability functions and cast into a clustering problem. It can be classified as forward and backward approaches [107]. In the forward approach [108], the multiple point sets are assumed to be noisy observations of the mean point set. In the backward approach [2,3,109], the mean point set is assumed to be a noisy observation of multiple point sets. Both the forward and backward approaches consist of two steps:

- the construction of the mean point set.
- the estimation of the transformation between the multiple point sets and mean point set.

**Figure 5:**
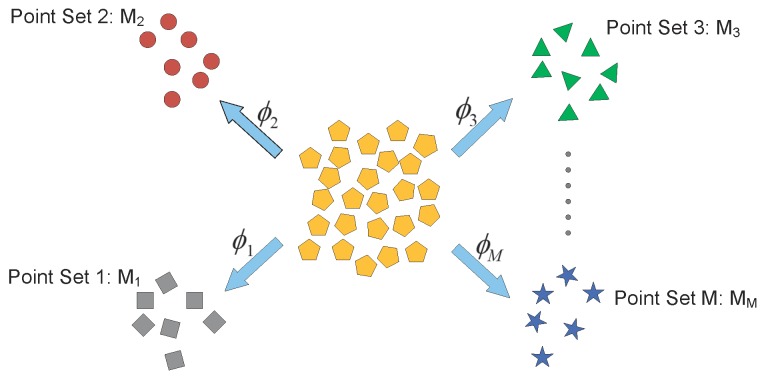
The forward approach of groupwise point set registration method in [108].

These two steps are iteratively computed to register the multiple point sets. In [108], the forward approach of groupwise point set registration method was proposed and it is shown schematically in Figure 5. It is assumed that the multiple point sets are noisy observations of mean point set:

(4) $$p\left(M_{ji}\right)=\sum_{k=1}^{K}\alpha_{k}N\left(M_{ji}\left|\phi_{j}\left(\Gamma_{k}\right),\Omega_{k}\right.\right)$$

where $\Gamma_{k}$ and $\Omega_{k}$ are the mean vector and covariance matrix, respectively; $\alpha_{k}$ is the mixing coefficient; and $\phi_{j}\left(.\right)$ is the transformation function for the forward approach. $\Gamma_{k}$ is assumed as the mean point set in the forward approach. In [108], the EM algorithm was proposed to estimate the mean point set and the parameters in the transformation function.

**Figure 6:**
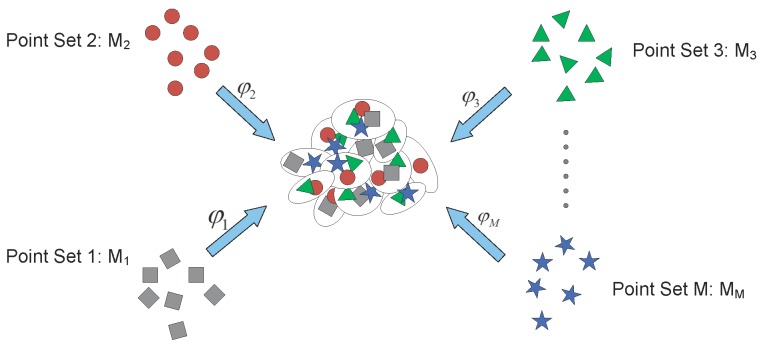
The backward approach of groupwise point set registration method in [2].

In [2,3], the backward approach of groupwise point set registration method was developed and it is shown schematically in Figure 6. It is assumed that the multiple point sets are transformed realizations of mean point set:

(5) $$p\left(M_{ji}\right)=\sum_{k=1}^{K}\beta_{k}N\left(\varphi_{j}\left(M_{ji}\right)\left|\Upsilon_{k},\Xi_{k}\right.\right)$$

where $\Upsilon_{k}$ and $\Xi_{k}$ are the mean vector and covariance matrix, respectively; $\beta_{k}$ is the mixing coefficient; and $\varphi_{j}\left(.\right)$ is the transformation function for the backward approach. The $\Upsilon_{k}$ is assumed as the mean point set in the backward approach. The EM algorithm is also used to register the multiple point sets simultaneously. Table 1 summarizes some representative methods for point set registration.

## 4. Experiments

In this section, 10 representative point set registration algorithms are selected to conduct some experiments. These 10 representative methods are as follows: ICP (Available at http://www.cvlibs.net/software/libicp/) [110], TPS-RPM (Available at https://www.cise.ufl.edu/~anand/publications.html) [35], KC (Available at http://www.cs.cmu.edu/~ytsin/KCReg/) [36], CPD (Available at https://sites.google.com/site/myronenko/research/cpd) [25], CPD-GL (Available at https://sites.google.com/site/jiayima2013/) [1], SCGF (Available at https://sites.google.com/site/2013gwang/SCGF.zip) [17], CDF-HC (Available at https://www.cise.ufl.edu/~anand/publications.html) [105], Rényi’s second order entropy (Rényi’s) [106], Student’s t-mixture model (TMM) [111], and groupwise probability-based method (JRMPC) (Available at https://team.inria.fr/perception/research/jrmpc/) [2,3]. The ICP, TPS-RPM, KC, CPD, CPD-GL, and SCGF are pairwise point set registration methods. The CDF-HC, Rényi’s, TMM, and JRMPC are groupwise point set registration methods. The performance of these representative point set registration algorithms is validated on the toy data sets from [35]. The validation considers different levels of noise, to object deformation, rotation, and occlusion. To evaluate the performance of the rivals, the cost function of the optimization problem defined in (1), that is the Mean Squared Error Distance (MSED), is used. These representative point set registration algorithms were implemented compared in Matlab. The transformed function in the TPS-RPM, CPD, CPD-GL, and SCGF are chosen using a nonrigid transformation. The parameters in these representative algorithms are set as in the original papers. Each algorithm is carried out until it is converged or runs at least 50 iterations.

**Figure 7:**
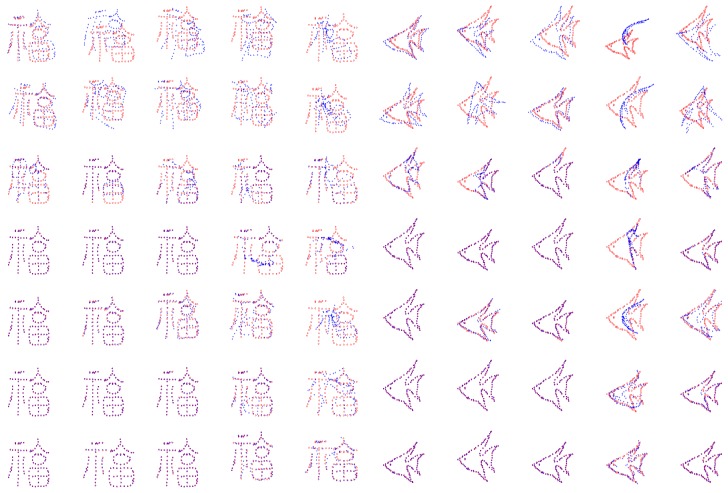
Registration results obtained from the application of pair-wise rivals on the Chinese characters set and fish shapes for different level of degradation. Data with different levels of deformation (first row) and the corresponding obtained results by KC (second row), ICP (third row), TPS-RPM (fourth row), CPD (fifth row), CPD-GL (sixth row), and SCGF (seventh row).

**Figure 8:**
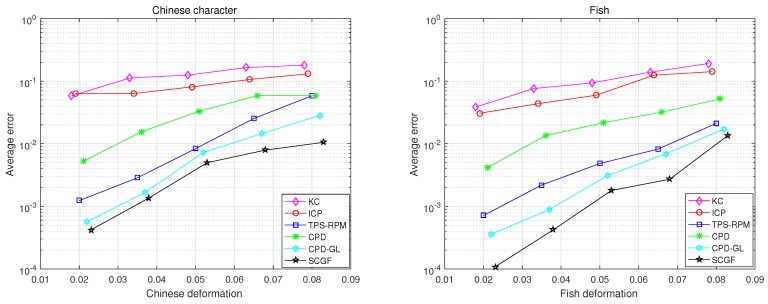
MSED, in log-scale, achieved from the application of the under-comparison pair-wise registration algorithms on the Chinese characters set and fish shapes for different levels of degradation.

In the pairwise point set registration experiments, the fish and Chinese character datasets [35] are considered. The qualitative results of these pairwise point set registration algorithms are given in Figure 7. It is observed that most algorithms can register under deformation degradations. The distance metrics for correspondence matching of these pairwise point set registration algorithms under varying deformation in fish and Chinese dataset are shown in Figure 8. It is observed that the SCGF has better registration accuracy performance than other algorithms. The average runtime of these algorithms are given in Table 2, which illustrates that the CPD is computationally most efficient.

**Figure 9:**
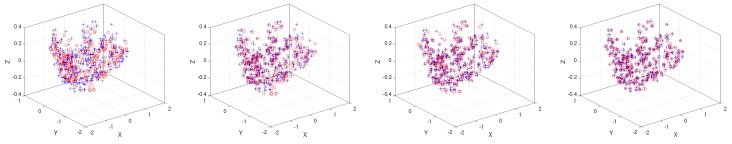
Registration results obtained from the application of pair-wise rivals on a specific example generated from 3D COPD data. Initial unregistered point sets (first column) and the results of CPD (second column), CPD-GL (third column), and SCGF (fourth column).

**Figure 10:**
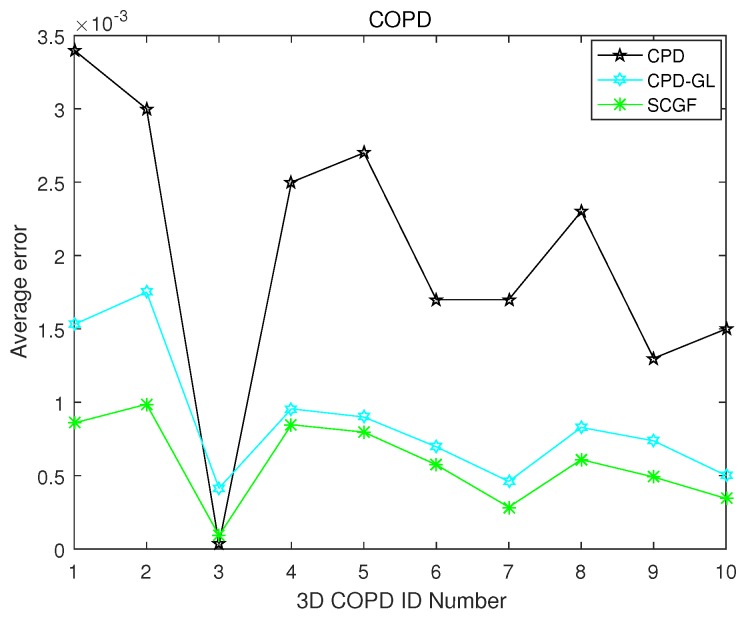
MSED achieved from the application of the under-comparison pair-wise registration algorithms on multiple point set groups generated by 3D COPD data (please see text for the details).

The 3D COPD data http://www.dir-lab.com/index.html is employed here. The “COPDID_300_iBH_xyz” is chosen as the model point set, while “COPDID_300_eBH_xyz” is considered as the scene point set. Thus, ten pairs of point sets are generated, where “ID” ∈[1,10]. The example of the registration results of pairwise point set registration algorithms on “COPD1_300_iBH_xyz” vs. “COPD1_300_eBH_xyz” is depicted in Figure 9, which demonstrates that SCGF and CPD-GL can register the 3D COPD point set. Furthermore, the comparison results using registration error are illustrated in Figure 10. It can be observed that the CPD-GL has almost same registration accuracy performance with SCGF.

In brief, the SCGF algorithm have more accuracy, while it needs more computational effort. The CPD-GL algorithm has almost same registration accuracy performance with SCGF, but it has a lower computation load than SCGF algorithm. Therefore, the CPD-GL algorithm has the tradeoff between accuracy and computational complexity.

**Figure 11:**
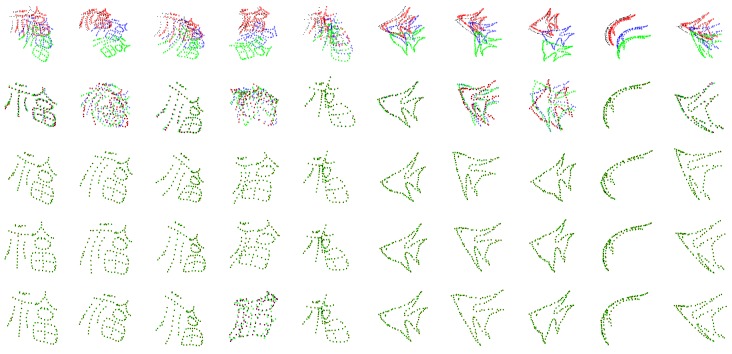
Registration results obtained from the application of group-wise rivals on the Chinese characters set and fish shapes for different level of degradation. Data with different levels of deformation (first row) and the corresponding obtained results by CDF-HC (second row), JRMPC (third row), TMM (fourth row), and Rényi’s (fifth row).

**Figure 12:**
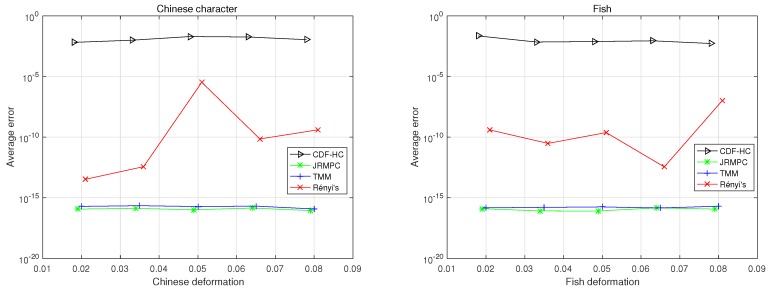
MSED, in log-scale, achieved from the application of the under-comparison group-wise registration algorithms on the Chinese characters set and fish shapes for different levels of degradation.

In the groupwise point set registration experiments, the fish and Chinese character datasets are also considered and there are four-point sets. To generate the multiple point set groups, parameters of deformation in a rigid transformation are chosen uniformly in the following range: [0.02, 0.08]. The qualitative results of the groupwise point set registration experiments are shown in Figure 11. The distance metrics for correspondence matching of these groupwise point set registration algorithms under varying deformation in fish and Chinese dataset are depicted in Figure 12. From the Figure 11 and Figure 12, it can be observed that JRMPC, TMM, and Rényi’s algorithms can register under deformation degradations. The average runtime of these algorithms are given in Table 3, which illustrates that the JRMPC is the computationally most efficient.

**Figure 13:**
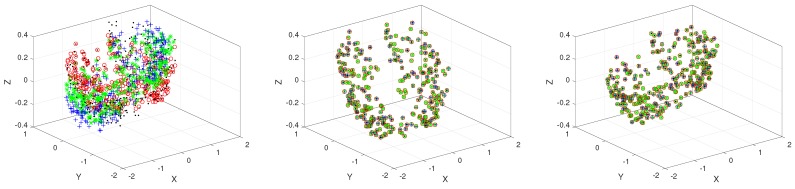
Registration results obtained from the application of group-wise rivals on a specific example generated from 3D COPD data. Initial unregistered point sets (first column) and the results of JRMPC (second column) and TMM (third column).

**Figure 14:**
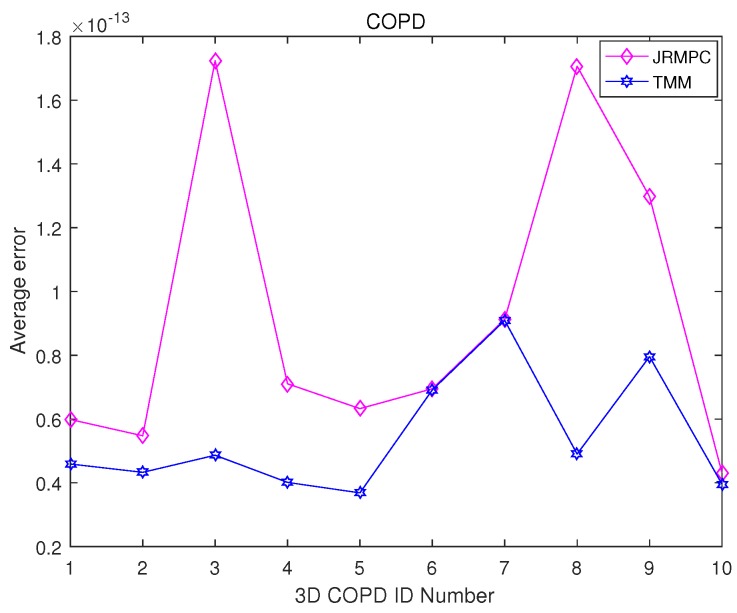
MSED achieved from the application of the under-comparison group-wise registration algorithms on multiple point set groups generated by 3D COPD data (please see text for the details).

Then, multiple 3D COPD point set groups are generated. It has ten point set groups, where each group has four point sets. The four point sets in each group are generated, where deformation parameters in a rigid transformation are chosen uniformly in the following range: [0.02, 0.08] on “COPDID_300_eBH_xyz”, where “ID” ∈[1,10], respectively. The example of the registration results of JRMPC and TMM algorithms on COPD data are depicted in Figure 13, which demonstrates that JRMPC and TMM algorithms can register this point set group. The statistics for the compared results are given in Figure 14, which unfolds that JRMPC algorithm has almost the same performance with the TMM algorithm.

Thus, from the results in the point set group experiment, the JRMPC and TMM algorithms present almost the same registration accuracy performance, but the JRMPC has lower computational load.

## 5. Conclusions

This paper presents a review of the state-of-the-art point set registration methods. From the pairwise point set registration to groupwise point set registration, the modeling methods are discussed with a summary of their pros and cons. In the pairwise point set registration, the point set registration methods can be classified as distance-based methods, filtering-based methods and probability-based methods. In the groupwise point set registration, the point set registration methods have been classified as information theoretic-based methods and probability-based methods. Some evaluation metrics to evaluate the performance of point set registration are given. Furthermore, several experiments with some representative point set registration algorithms are performed. From the numerical experiments, the CPD-GL pairwise registration [1] and the JRMPC groupwise registration algorithms [2,3] offer a tradeoff between accuracy and computational burden.

Although many methods have been proposed for point set registration, there are still many challenges necessary for further study:

(1) Object localization for the purpose of autonomous vehicles and in health systems requires point set registration over massive and high-dimensional point sets. One direction for alleviating this problem relies on point or feature selection. Clustering algorithms can then be used to cope with such challenges. Sparse Bayesian learning methods are also capable of identifying the suitable features for point set registration.
(2) There is a need for more benchmark examples and large scale datasets with ground truth for thorough performance evaluation of the developed approaches.
(3) Point set registration is an essential step towards target tracking and pattern recognition. There is a scope of assessing its impact on the entire monitoring system of interest, with different levels of autonomy.

## Figures and Tables

**Table 1:** The representative methods for point set registration.

Research Study	Pairwise/Groupwise	Method	Rigid/Non-Rigid	Parametric/Non-Parametric Model	Characteristics
Besl and McKay [23]	Pairwise	Distance-based method	Rigid	Parametric Model	(1) Sensitive to the initialization (2) Trapping into local minima
Gold et al. [34]	Pairwise	Distance-based method	Rigid	Parametric Model	(1) Combining deterministic annealing and softassign optimization (2) Restricting to perform the rigid-body transformation
Chui et al. [35]	Pairwise	Distance-based method	Non-rigid	Parametric Model	Difficult to extend to perform higher dimension
Tsin et al. [36]	Pairwise	Distance-based method	Rigid and Non-rigid	Parametric Model	Maximizing the KC of point sets
Jian et al. [38]	Pairwise	Distance-based method	Rigid and Non-rigid	Parametric Model	Minimizing the Euclidean distance of two GMMs
Leordeanu et al. [46]	Pairwise	Distance-based method	Rigid and Non-rigid	Non-Parametric Model	Convexifying the QAP problem by spectral relaxation method
Cour et al. [47]	Pairwise	Distance-based method	Rigid and Non-rigid	Non-Parametric Model	Convexifying the QAP problem by semidefinite-programming relaxation
Almohamad et al. [50]	Pairwise	Distance-based method	Rigid and Non-rigid	Non-Parametric Model	Convexifying the QAP problem by doubly stochastic relaxation
Zhou et al. [22]	Pairwise	Distance-based method	Rigid and Non-rigid	Non-Parametric Model	Factorizing the large pairwise affinity matrix into some smaller matrices
Sandhu et al. [67]	Pairwise	Filter-based method	Rigid	Non-Parametric Model	Using a particle filter to register the point sets
Li et al. [16]	Pairwise	Filter-based method	Rigid	Non-Parametric Model	(1) Using a cubature Kalman filter to register the point sets (2) The correspondence should be computed in advance
Myronenko et al. [25]	Pairwise	Probability-based method	Rigid and Non-rigid	Parametric Model	(1) Using a GMM model to formulate the distribution of the point sets (2) Maximizing the likelihood of GMM
Ma et al. [76]	Pairwise	Probability-based method	Rigid and Non-rigid	Parametric Model	Developing a locally linear transforming for local structure constrict
Wang et al. [104]	Groupwise	Information theoretic measure	Rigid and Non-rigid	Parametric Model	Proposing a CDF-JS divergence as the cost function
Chen et al. [105]	Groupwise	Information theoretic measure	Rigid and Non-rigid	Parametric Model	Developing a CDF-HC divergence as the cost function
Giraldo et al. [106]	Groupwise	Information theoretic measure	Rigid and Non-rigid	Parametric Model	Using a Rényi’s second order entropy divergence as the cost function
Rasoulian et al. [108]	Groupwise	Probability-based method	Non-rigid	Parametric Model	Assumed that the multiple point sets are the noisy observations of mean point set
Evangelidis et al. [2,3]	Groupwise	Probability-based method	Rigid	Parametric Model	Assumed that the multiple point sets are transformed realizations of mean point set

**Table 2:** Runtime of pairwise point set registration algorithms on different datasets.

Method	KC	ICP	TPS-RPM	CPD	CPD-GL	SCGF
Fish	0.41 s	0.47 s	2.37 s	0.22 s	0.42 s	37.04 s
Chinese	0.39 s	0.50 s	2.38 s	0.22 s	0.73 s	27.64 s

**Table 3:** Runtime of groupwise point set registration algorithms on different datasets.

Method	CDF-HC	JRMPC	TMM	Rényi’s
Fish	58.34 s	20.06 s	27.11 s	60.06 s
Chinese	77.70 s	20.17 s	31.70 s	72.72 s
